# Asymmetric localization of *Arabidopsis *SYP124 syntaxin at the pollen tube apical and sub-apical zones is involved in tip growth

**DOI:** 10.1186/1471-2229-10-179

**Published:** 2010-08-18

**Authors:** Pedro Ângelo Silva, Reiaz Ul-Rehman, Cláudia Rato, Gian-Pietro Di Sansebastiano, Rui Malhó

**Affiliations:** 1 Universidade de Lisboa, Faculdade de Ciências de Lisboa, BioFIG, 1749-016 Lisboa, Portugal; 2 Di.S.Te.B.A., Università del Salento; via prov. Lecce-Monteroni; 73100 - Lecce, Italy; 3 Institute of Medical Sciences, School of Medical Sciences, University of Aberdeen, Foresterhill, Aberdeen, AB25 2ZD, UK

## Abstract

**Background:**

The continuous polarized vesicle secretion in pollen tubes is essential for tip growth but the location of endo- and exocytic sub-domains remains however controversial. In this report we aimed to show that *Arabidopsis thaliana *syntaxins are involved in this process and contribute to spatially define exocytosis and membrane recycling.

**Results:**

Using GFP-fusion constructs, we imaged the distribution of pollen-specific (AtSYP124) and non-pollen syntaxins (AtSYP121 and AtSYP122) in transiently transformed *Nicotiana tabacum *pollen tubes. All three proteins associate with the plasma membrane and with apical vesicles indicating a conserved action mechanism for all SYPs. However, the GFP tagged SYP124 showed a specific distribution with a higher labelling at the plasma membrane flanks, 10-25 μm behind the apex. This distribution is affected by Ca^2+ ^fluxes as revealed by treatment with Gd^3+ ^(an inhibitor of extracellular Ca^2+ ^influx) and TMB-8 (an inhibitor of intracellular Ca^2+ ^release). Both inhibitors decreased growth rate but the distribution of SYP124 at the plasma membrane was more strongly affected by Gd^3+^. Competition with a related dominant negative mutant affected the specific distribution of SYP124 but not tip growth. In contrast, co-expression of the phosphatidylinositol-4-monophosphate 5-kinase 4 (PIP5K4) or of the small GTPase Rab11 perturbed polarity and the normal distribution of GFP-SYP but did not inhibit the accumulation in vesicles or at the plasma membrane.

**Conclusions:**

The results presented suggest that in normal growing pollen tubes, a net exocytic flow occurs in the flanks of the tube apex mediated by SYP124. The specific distribution of SYP124 at the plasma membrane is affected by changes in Ca^2+ ^levels in agreement with the importance of this ion for exocytosis. Apical growth and the specific localization of SYP124 were affected by regulators of membrane secretion (Ca^2+^, PIP5K4 and Rab11) but competition with a dominant negative mutant affected only SYP distribution. These data thus suggest that syntaxins alone do not provide the level of specificity that is required for apical growth and that additional signalling and functional mechanisms are required.

## Background

Pollen tube growth and reorientation occur only at the extreme apex of the tube due to polarized fusion of secretory vesicles, which transport cell wall components to the growing tip [1,2]. This exocytic delivery of material to the extending apex is found in root hairs [3], fungal hyphae [4] and rhizoids [5], all tip-growing cells. In pollen tubes, the membrane material provided by vesicles that fuse with the plasma membrane was calculated to exceed the needs to maintain growth rates suggesting an underlying recycling process [2,6]. It was generally assumed that exocytosis events occur mostly at the extreme apex while membrane recycling (endocytosis) would take place further back from the tip, at the flanks of the apex and/or at sub-apical regions. Several lines of evidence supported this hypothesis: (a) higher Ca^2+ ^values at the extreme apex [1] which could favour exocytosis; (b) a higher concentration of pectin methylesterases [7], Sec-GFP and AGP-GFPs [8] reported at the extreme apex; (c) the presence of an exocyst complex also reported at the extreme tip [9]; and (d) the absence of clathrin-coated vesicles from the extreme apex in pollen tubes preserved by freeze-fixation [10]. However, some of these data only indirectly concerned secretion or were obtained with methods that precluded a proper analysis of vesicle dynamics. The use of FM styryl dyes (FM1-43 and FM4-64) helped to generate a picture of vesicle and membrane dynamics during apical growth [2,11] but no evidence is available at present to actually prove that these labels correspond to that of vesicles destined for exocytosis [12]. It nevertheless allowed us to generate other working hypothesis namely the existence of a rapid endocytosis (or kiss-and-run) mechanism at the extreme apex coupled to a "conventional" mechanism with clathrin-mediated endocytosis occurring at sub-apical regions [12,13].

In this work we tried to further dissect the secretory process by investigating the distribution and role of syntaxins during pollen tube growth. The syntaxins are a large evolutionarily conserved family of proteins required for docking and fusion of transport vesicles in eukaryotic cells. Individual syntaxins reside on the organelles of the endomembrane system in which it is believed that they assemble with other proteins of the SNARE (for soluble N-ethylmaleimide sensitive factor attachment protein receptor) family to form SNARE complexes [14]. Syntaxins are classified on a structural base as Qa-SNAREs, they interact on the target membrane with two partners classified as Qb- and Qc-SNAREs [15]. The SNARE complex on the target membrane (also known as t-SNARE complex) serves as a binding site for R-SNAREs on the transport vesicle (for this reason also called v-SNAREs) and assures docking of the vesicle to target membrane driving membrane fusion [16]. Mapping of these proteins in live cells can thus help to pinpoint exocytic events and their dynamics. Using GFP-fusion proteins, we analysed the distribution of SYP124, a pollen-specific syntaxin [Microarray Database Genevestigator, GeneChip; [17]], during pollen tube growth and upon modulation by inhibitors and regulatory proteins. Although highly conserved, some research has suggested that individual members of particular gene families have distinct localizations and thus may have distinct functions [15,18,19]. So in order to test how specific is SYP localization, we also transformed pollen with GFP-tagged, non-pollen specific, SYP121 and SYP122. All three SYPs belong to the same sub-family of Qa-SNAREs, a family of 17 members with broad distribution inside the cell [20,21]. SYP121 was reported to be involved in ABA-related secretion [22] while SYP122 seems to have a more general function in secretion [23], including a role in cell wall deposition [24]. Here we report that GFP-SYP124 showed a specific distribution with a higher labelling at the plasma membrane flanks, 10-25 μm behind the apex of growing pollen tubes and that this distribution is affected by regulators of membrane secretion (Ca^2+^, PIP5K4 and Rab11).

## Results

To study the distribution of syntaxins during pollen tube growth, pollen of *Nicotiana tabacum *was transiently transfected with a GFP-AtSYP124 fusion construct with the chimera cloned downstream of the Lat52 promoter [25]. This strategy had already been successfully used in previous studies [e.g. [26,27]]. Tobacco pollen is easily transformed using biolistics, the tubes are large, grow fast and they are more tolerant to the laser irradiation required for imaging (when compared to pollen from *Arabidopsis*). At*SYP124 *is a pollen-specific gene [Microarray Database Genevestigator, GeneChip; [17,28]]) from a highly conserved family. To investigate if this specificity is translated into a specific distribution or if syntaxin distribution simply follows the cell's secretory requirements, we also transformed pollen with GFP fusion constructs of AtSYP121 and AtSYP122, non-pollen syntaxins that have been previously characterized in protoplasts by us [23] and with dominant-negative (DN) versions of all three proteins.

### Syntaxins accumulate in apical vesicles and at the membrane flanks of the growing pollen tube

**Figure 1:**
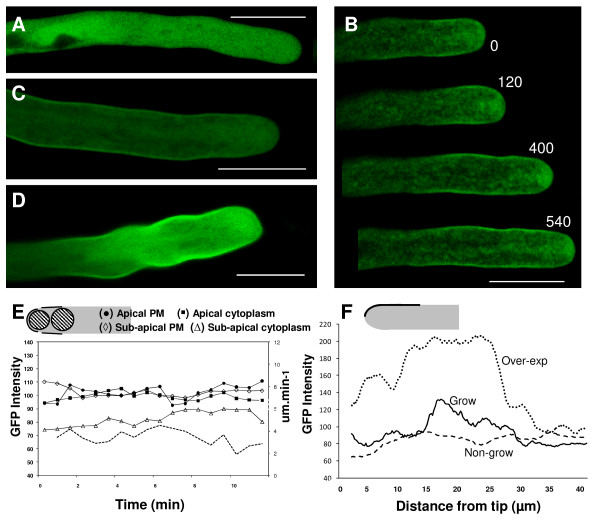
**Confocal imaging of *N. tabacum *pollen tubes expressing GFP-AtSYP124**. Scale bars = 20 μm. **A**: Optical section of a growing pollen tube transformed with free GFP showing a cytosolic distribution. **B**: Time-course series of a growing tobacco pollen tube transiently transformed with the GFP-SYP124 construct showing the subcellular localization of SYP124 (observation made ~6 h after transformation). The protein seemed to accumulate mostly in apical vesicles and at the flanks of the plasma membrane near the pollen tip. Numbers refer to the time interval (in seconds). Images are representative of twelve independent experiments. **C**: Optical section of a non-growing pollen tube transformed with GFP-SYP124. **D**: Pollen tube expressing high levels of GFP-SYP124 exhibited disturbed growth and perturbations in apical morphology. The protein accumulates at the flanks but also at the apical plasma membrane. **E**: Time course analysis of GFP fluorescence intensity in the growing pollen tube shown in B. Each line is representative of measurements of the fluorescent signal made in the lines/circles depicted in the diagram for 12 different pollen tubes: apical plasma membrane (black circle); apical cytoplasm (black square); sub-apical plasma membrane (lozenge); sub-apical cytoplasm (triangle). Growth rate measurements (μm.min^-1^) are displayed by the dashed line. **F**: Distribution of average fluorescence intensity along the first 40 μm of the pollen tubes plasma membrane as depicted in the diagram for growing (Grow - solid line; n = 12), non-growing (Non-grow - dashed line; n = 3) and pollen tubes over-expressing GFP-SYP124 (Over-exp - dotted line; n = 5). Differences are statistically significant (P = 5E^-9^). Error bars not displayed for the sake of clarity.

In actively growing pollen tubes (growth rate of at least 3 μm.min^-1^) imaged 5-6 h after transformation, free GFP showed a uniform distribution throughout the cytoplasm (figure 1A). In cells expressing GFP-SYP124 (n = 12), the fluorescence mainly localized to the plasma membrane with a higher signal at the flanks of the pollen tube (10-25 μm behind the extreme tip) and in the characteristic inverted cone of apical vesicles (figure 1B, E, F). When pollen tubes slowed down or ceased to grow, fluorescence was still visible associated to the plasma membrane but the higher apical vesicle labelling dissipated (figure 1C). Over-expression of higher levels of GFP-SYP124 (observations made 7-9 h after transformation - figure 1D) led to a significantly higher amount of fluorescence associated with the plasma membrane (figure 1F) and perturbations in apical morphology (changes in tip diameter) but not to a significant disturbance of polarity. As recently described by McKenna *et al. *[29] using phase-correlation analysis, we frequently observed that higher growth rates correlated with higher signals in the sub-apical membrane (when compared to the extreme apical membrane)(figure 1E).

**Figure 2:**
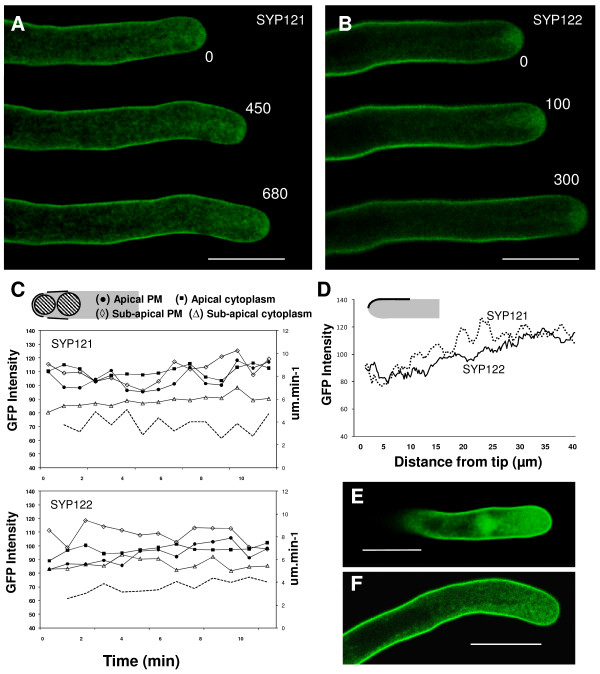
**Confocal imaging of *N. tabacum *pollen tubes expressing GFP-AtSYP121 and GFP-AtSYP122**. Scale bars = 20 μm. **A and B**: Time-course series of growing tobacco pollen tubes transiently transformed with the GFP-SYP121 (**A**) and GFP-SYP122 (**B**) constructs (observations made ~6 h after transformation). Both proteins seem to accumulate similarly, mostly in apical vesicles and at the plasma membrane near the pollen tip. Numbers refer to the time interval (in seconds). **C**: Time course analysis of GFP fluorescence intensity in the growing pollen tubes shown in A and B. Imaging and measurements were performed under the same conditions and settings as in figure 1E. **D**: Distribution of average fluorescence intensity along the first 40 μm of the pollen tubes plasma membrane as depicted in the diagram for growing pollen tubes expressing GFP-SYP121 (dotted line; n = 9) and GFPSYP122 (solid line; n = 7). The distribution of SYP121 and SYP122 are significantly different from GFP-SYP124 (P = 9E^-39 ^and P = 2E^-25 ^respectively) with the region 10-25 μm behind the apex not showing the highest labelling. Error bars not displayed for the sake of clarity. **E and F**: Pollen tubes expressing high levels of GFP-SYP121 (**E**) and GFP-SYP122 (**F**) exhibited disturbed protein localization but no perturbations in apical morphology.

Observations made in cells expressing the non-pollen SYP121 (n = 9) and SYP122 (n = 7) revealed also GFP-chimeras localization on the plasma membrane (figure 2A-C) in agreement with the highly conserved structure of these proteins (Additional file 1). The distribution pattern of the non-pollen SYP was nevertheless distinguishable from that of GFP-SYP124, with changes that could justify the specific expression profile in different tissues. The fluorescence distribution at the plasma membrane, even at regions further back from the sub-apex (25-40 μm behind the extreme tip), was more uniform with SYP121 and SYP122 (when compared to their pollen homologue - figures 1F and 2D). This pattern was more similar to the labelling provided by FM1-43 and FM4-64 dyes [[30], additional file 2], indicative of less specific sorting. Higher levels of either GFP-SYP121 or GFP-SYP122 led to saturation of plasma membrane fluorescence but to no changes in pollen morphology (figure 2E,F).

### Syntaxin localization at membranes is influenced by [Ca^2+^]_c _levels

**Figure 3:**
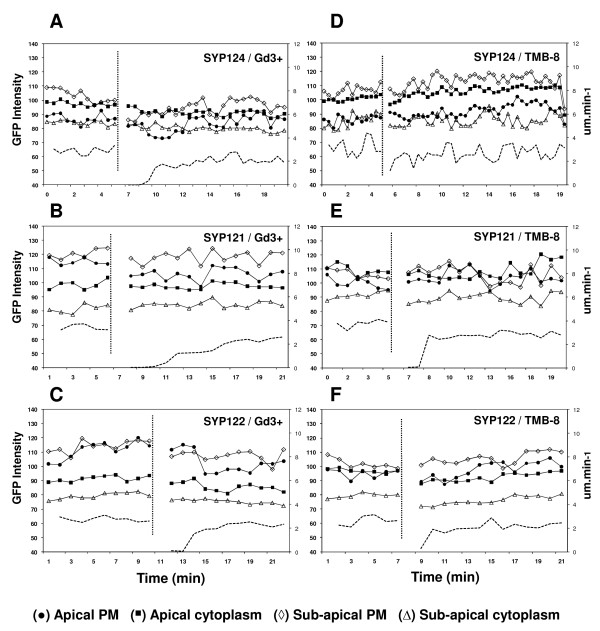
**Time course analysis of GFP fluorescence intensity in *N. tabacum *growing pollen tubes transformed with GFP-SYPs (124, 121, 122) and exposed to 20 μM of extracellular Gd**^**3+ **^**(A-C) or TMB-8 (D-F)**. Imaging and measurements were performed under the same conditions and settings as in figure 1E. Growth rate measurements (μm.min^-1^) are displayed by the dashed lines. Addition of the inhibitors was performed at the time indicated by the vertical dotted line and collection of data resumed after inhibitor wash out. Each graph is representative of 5 independent pollen tubes. Symbols indicate measurements performed in the apical plasma membrane (black circle), apical cytoplasm (black square), sub-apical plasma membrane (lozenge) and sub-apical cytoplasm (triangle) according to the diagram in figure 1E.

To test if the sub-cellular localization of syntaxins was affected by changes in [Ca^2+^]_c _levels (a known modulator of growth and exocytosis), pollen tubes expressing GFP-fusion constructs of SYP124, 121 and 122 were imaged before and after addition of 20 μM gadolinium chloride or 3,4,5-trimethoxybenzoic acid 8-(diethylamino)octyl ester (TMB-8). Gadolinium (Gd^3+^) is an inhibitor of plasma membrane cationic channels, preventing Ca^2+ ^influx, while TMB-8 inhibits intracellular Ca^2+ ^mobilization [31]. At this concentration both inhibitors transiently reduced growth rate but allowed fast recovery upon wash-out (figure 3).

Gd^3+ ^had a similar effect in the localization pattern of all three syntaxins. In pollen tubes expressing GFP-SYP124, along with a reduction in growth rate, the addition of this ion to the extracellular medium caused a transient but significant (≥ 5%) reduction in the association of the fluorescent signal with the apical (P < 0,01) and sub-apical plasma membrane (P < 0,001) (figure 3A; n = 5). However, the signal associated with both apical and sub-apical cytoplasmic regions exhibited no significant changes (< 5%; P > 0,01). The fluorescent signal of SYP121 and SYP122 was also found to reduce upon addition of Gd^3+ ^(figure 3B-C; n = 10) but in contrast to SYP124, the reduction was below our significance threshold. This suggests that influx of extracellular Ca^2+ ^is important for specific SYP localization (and accumulation) at the plasma membrane but not to their trafficking through secretory vesicles. We further quantified if the reduced accumulation at the plasma membrane upon addition of Gd^3+ ^was higher in the apical or sub-apical region. In all three syntaxins, the reduction was found to be slightly higher in the sub-apical region but this difference was also below our significance threshold.

TMB-8 had a reduced effect in the localization of all three syntaxins (figure 2D-F; n = 15). Its addition caused a transient reduction in plasma membrane and cytoplasmic fluorescence for SYP124, 121 and 122 but changes were non significant (≤5%) in contrast with the inhibition of growth rates. These observations suggest that the effects are likely to be a consequence of growth rate inhibition and not directly of intracellular Ca^2+ ^modulation.

### Syntaxins localization is regulated by signalling pathways

The recent modelling of Kato *et al. *[28] suggests that, although essential, syntaxins play more of a structural than a regulatory role in the secretory process. To further investigate this we devised a set of experiments where syntaxins were co-expressed with a truncated version consisting only in the N-terminal portion of SYP [23] or with known regulatory proteins of the secretory pathway (PIP5K4 [27,32] and Rab11 [8]).

**Figure 4:**
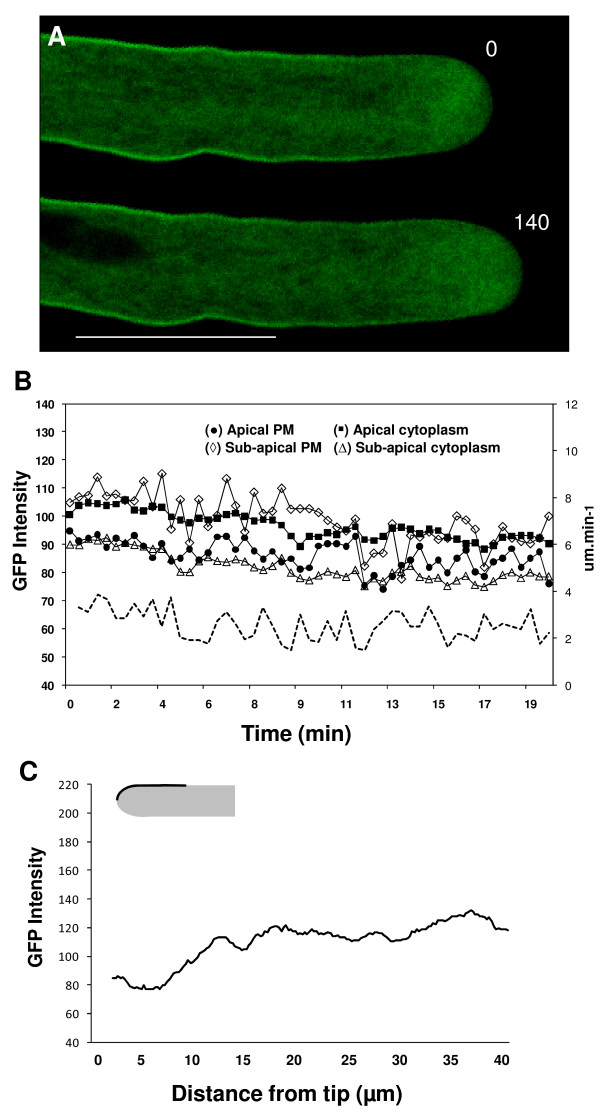
**Confocal imaging of *N. tabacum *pollen tubes co-expressing SYP124T (truncated protein lacking the transmembrane domain) with full GFP-SYP124**. **A**: Optical sections of a growing pollen tube. The cell exhibited no perturbations in apical morphology but protein localization exhibits a higher labelling in the plasma membrane further away from the sub-apical region (when compared to figure 1B). Numbers refer to the time interval (in seconds). Scale bar = 20 μm. **B**: Time course analysis of GFP fluorescence intensity in the first 20 μm of the pollen tube shown in A. Each line is representative of measurements of the fluorescent signal made in the lines/circles depicted in the diagram of figure 1E for 10 different pollen tubes. Growth rate measurements (μm.min^-1^) are displayed by the dashed lines. **C**: Distribution of average fluorescence intensity along the first 40 μm of the pollen tubes plasma membrane (n = 10) as depicted in the diagram. The distribution is significantly different (P = 3E^-54^) from GFP-SYP124 alone (compare with figure 1F) but very similar to GFPSYP121 and GFP-SYP122 (P = 0,01) (compare with figure 2D). Error bars not displayed for the sake of clarity.

The co-expression of full GFP-SYP124 with its truncated version (SYP124T) led to no visible change in pollen tube morphology or growth rate. However, the localization pattern of SYP124 at the plasma membrane shifted with regions further back from the sub-apex now more evidently labelled (figure 4A-C; n = 10). This pattern resembles the localization of the non-pollen SYPs (121 and 122; figure 2) and suggests a perturbation in the interaction of SYP124 with its targets. In agreement with a specificity issue, the localization pattern of SYP121 or 122 was not changed by the co-expression with their respective truncated versions (data not shown).

**Figure 5:**
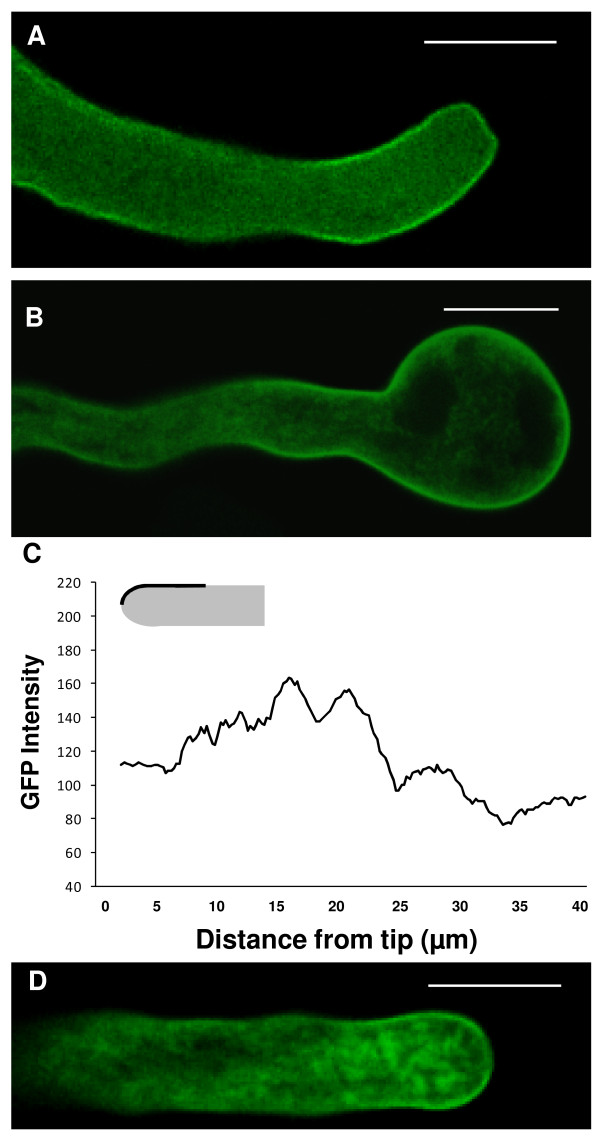
**Confocal imaging of *N. tabacum *pollen tubes co-expressing PIP5K4 with GFP-SYP124**. Scale bars = 20 μm. **A**: Optical section of a non-growing pollen tube due to PIP5K over-expression. The cell exhibits partial loss of polarity and high protein localization in the flanks and apical plasma membrane. **B**: Optical section of a depolarized pollen tube due to PIP5K over-expression. SYP124 distribution is almost uniform in the swollen apex. **C**: Distribution of average fluorescence intensity along the first 40 μm of the pollen tubes plasma membrane (n = 10) as depicted in the diagram. The distribution is statistically different (P = 3E^-31^) from GFP-SYP124 alone (compare with figure 1F). Error bars not displayed for the sake of clarity. **D**: Optical section of a halted pollen tube due to PIP5K over-expression. Along with high protein localization in the flanks and apical plasma membrane, labelling of large endomembrane-like structures is visible.

The co-expression of GFP-SYP124 with PIP5K4 led to a different scenario. It was recently shown that over-expression of PIP5K4, a protein that normally concentrates in the flanks of the pollen tubes, perturbs pollen tube morphology and affects secretion [27,32]. Here we found that co-expression (~7-9 h after transformation), along with a disturbance in polarity, caused changes in GFP-SYP124 localization (figure 5A-C; n = 10). Accompanying the partial loss of polarity, the association of GFP fluorescence with the apical plasma membrane increased, the typical higher labelling of the apical cytoplasm dissipated (figure 5A-C), and some cells (n = 4) exhibited large labelled structures in the cytoplasm that extended to the apical region (figure 5D). Similar results were obtained when co-expression was performed with GFP-SYP121 or with GFP-SYP122 (data not shown) and indicate a modulation of syntaxin trafficking and distribution by the phosphoinositide signalling pathway.

**Figure 6:**
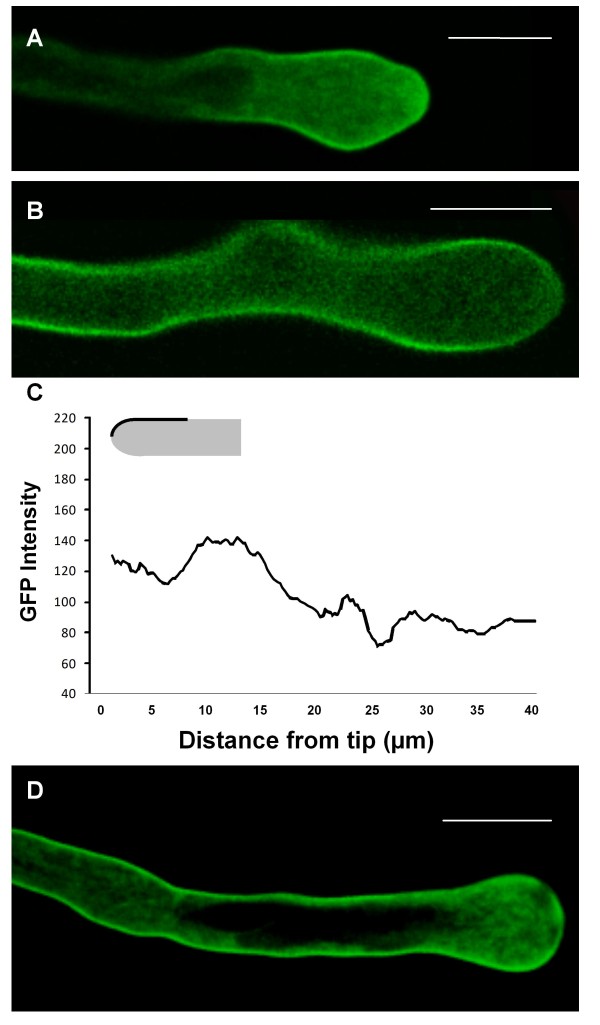
**Confocal imaging of *N. tabacum *pollen tubes co-expressing Rab11DN with GFP-SYP124**. Scale bars = 20 μm. **A and B**: Optical section of pollen tubes with disturbed apical morphology due to Rab11DN over-expression. Along with a partial loss of polarity, the cells exhibit high protein localization in the flanks and apical plasma membrane and almost complete dissipation of the apical vesicle labelling. **C**: Distribution of average fluorescence intensity along the first 40 μm of the pollen tubes plasma membrane (n = 10) as depicted in the diagram. The distribution is statistically different (P = 2E^-18^) from GFP-SYP124 alone (compare with figure 1F). Error bars not displayed for the sake of clarity. **D**: Optical section of a depolarized pollen tube due to Rab11DN over-expression. SYP124 distribution is uniform in the swollen apex. Along with high protein localization in the flanks and apical plasma membrane, labelling of large endomembrane-like structures is visible.

Syntaxin activity was also recently shown to be modulated by small GTPases of the Rab sub-family, namely Rab11 [23]. In pollen tubes, expression of a dominant-negative GDP-bound version of Rab11 was shown to induce depolarization of the growth axis and to disturb membrane trafficking [8]. Therefore we co-expressed a dominant-negative GDP-bound Rab11DN together with GFP-SYP124. This co-expression led to significant changes in pollen tube tip morphology, occasional depolarization and also to altered SYP localization (figure 6A-D; n = 10). As observed with the co-expression of PIP5K4, the association of SYP with the apical plasma membrane increased and the typical higher labelling of apical vesicles dissipated (figure 6A,C). Furthermore, the expression of the Rab11DN led to the labelling of large structures in the apex that could result from vesicle aggregation or large vesicles formation (figure 6D). Similar results were obtained when co-expression was performed with GFP-SYP121 or with GFP-SYP122 (data not shown).

## Discussion

### The use of GFP-syntaxin fusion constructs to analyse secretion in pollen tubes

Although essential for understanding pollen tube apical growth, the location and dynamics of exocytic events is still controversial [2,33]. GFP fusion proteins have been used linked to secreted proteins [8,7,29,34] so the localization reflects their accumulation and not necessarily the point of vesicle fusion [33]. Zonia and Munnik [11] took advantage of the complementary emission spectra of FM1-43 and FM4-64 to perform an elegant experiment where the pollen tubes were first labelled with one dye until the endomembrane compartments were saturated and all exocytic vesicles labelled. The authors then imaged the cells shortly after addition of the second dye; the assumption was that the later will label only endocytic vesicles. By comparing the two signals the authors suggested that exocytosis takes place in the flanks of the pollen tube while at the extreme apex endocytosis prevails. Although limited by the characteristics of the dyes, this hypothesis raises new questions concerning the localization of the exocytic machinery and vesicle dynamics in the pollen tube apex. Here we analysed the localization and dynamics of syntaxin proteins which are required for docking and fusion of secretory vesicles [16]. Expression of the pollen-specific SYP124 fused to GFP did not affect pollen tube growth rate or morphology and comparison with data obtained using non-pollen syntaxins (SYP121 and 122) revealed different and specific dynamics. As expected for such protein, the GFP fluorescent signal was strongly associated to the plasma membrane and secretory vesicles in the apical region thus strengthening the use of GFP-SYP124 as a marker for the study of secretory events in pollen tubes.

### Syntaxins accumulate at the plasma membrane flanks and in apical vesicles of the growing pollen tube

We found that GFP-tagged SYP124 mostly localizes to vesicles in the apical inverted cone and to the plasma membrane at the flanks of the growing pollen tube, 10-25 μm behind the tip. This pattern suggests that the preferential location for fusion is on a limited membrane domain at the sub-apical flanks and not at the extreme apex (additional file 2), in agreement with the recent hypothesis of an annulus shaped release zone [11,12]. The observation that membrane fluorescence extends to regions further back from the apex is likely explained by diffusion of the GFP-SYP124 when its ligands (yet to be identified) are saturated. When the tubes slowed down (or eventually stopped growing) the protein seemed to spread to the extreme apex of the tube and to regions further back from the sub-apical region. In a recent work, Enami *et al. *[21] also looked at the localization of AtSYP124 and showed its localization all over the plasma membrane and in the apical region. But in this case a critical interpretation is required because the work was done in *Arabidopsis *pollen, a system where is notoriously difficult to obtain proper imaging of growth and cell dynamics. Thus our option for using an heterologous system - tobacco pollen - where such limitations are minor and which have been used as a valid model to analyse the sub-cellular localization of Arabidopsis protein [26,27]. The data must be interpreted bearing in mind these advantages and limitations.

Imaging of the non-pollen SYP121 and SYP122 showed significant differences from SYP124 in the wider labelling of plasma membrane regions. Given the high degree of conservation in this protein sub-family, the data suggests that tissue-specificity may be relevant to assign interaction with membrane targets and not to determine rates of vesicle fusion. This hypothesis gain support by the co-expression experiments of SYP124 with its truncated version deleted of the transmembrane domain but still able to interact with the other partners of the SNARE complex [23]. This co-expression resulted in a less confined accumulation of SYP124 at the plasma membrane. In other experimental systems (e.g. protoplasts), the co-expression of truncated soluble variant of SYPs induced an alteration of the corresponding GFP-tagged full-length protein distribution pattern [23,35]. This was due to the dominant negative potential of the truncated form that competes with the native protein for the SNARE partner but has minimal impact on the formation of functional complexes [36]. Consequently, the "pool" of interactors with the non-mutated SYP is reduced and the protein will flow on the membrane differently.

### Syntaxins play a structural role and are modulated by multiple signalling pathways

Calcium is a known regulator of membrane secretion and of pollen tube growth [1,13]. SNARE fusion is inherently Ca^2+^-insensitive but an increase in Ca^2+ ^enhances SNARE-mediated liposome fusion [37]. We thus tested if the labelling intensity of SYP localization was affected by changes in the extracellular influx or intracellular release of Ca^2+^. In such experiments we resorted to low concentrations of specific chemical inhibitors that have been widely tested in plant cells [31]. But the results must nevertheless be interpreted with caution as one cannot rule out the possibility of side effects. When influx was partially blocked, a transient reduction was observed in the SYP124 fluorescence associated to the plasma membrane consistent with a reduction in fusion rates but continued membrane recycling. When we instead blocked the intracellular release of Ca^2+^, changes in SYPs distribution at the plasma membrane were not significant in agreement with recent findings that exocytosis is not profoundly affected by intracellular [Ca^2+^]_c _[29].

Consistent with previous finding that PIP5K4 modulates secretion and membrane recycling [27,32] we found that co-expression of PIP5K4 leads to altered distribution of SYPs. Namely, SYP124 is displaced and accumulates at the extreme apex and also in large endomembrane structures. This is in agreement with previous reports that PtdIns(4,5)P_2 _(the end product of PIP5K4 activity) helps to mark the plasma membrane as the appropriate target for vesicle fusion [38] and that PtdIns(4,5)P_2 _levels are known to positively correlate with the pool of secretory vesicles [39].

A similar effect was observed upon co-expression with a Rab11 DN mutant which was reported to modulate exocytosis in protoplasts [23] and to be crucial for tip growth in pollen tubes [8]. The observed effect of the Rab11DN confirms the role of these small Rab GTPases in the targeting of vesicles to the plasma membrane. Taken together these data suggests that syntaxins play mostly a structural role in pollen tube growth rather than specifying the rate of vesicle delivery. Their turnover and localization most likely follows the changes triggered by the multiple signalling pathways acting on pollen tube secretion. The data also agrees with the theoretical predictions of Kato *et al. *[28] that a knockout of *SYP*124 will not affect tube length; only a double knockout of *SYP*124 and *SYP*125 (the other pollen-specific gene coding for a syntaxin reported to have asymmetric localization - [21]) will have an effect. However, it should be noted that syntaxins are only one element of the complex machinery required for vesicle fusion and that other factors might be limiting the effect of SYP124. Additional work is required to characterize the function of syntaxins in plant fertilization.

## Conclusions

It was recently proposed that a rapid endocytosis mechanism might occur in the apex of rapidly growing pollen tubes [2,12,40]. This mechanism is a Ca^2+^-dependent process, coupled to exocytosis which does not require clathrin [41,42]. This rapid endocytosis could operate in parallel to a system where endo- and exocytosis are uncoupled. The uncoupling would be favoured when cell growth is arrested or it is slowed down (apical [Ca^2+^]_c _is lower) as it occurs in situations where the cell needs to interpret extracellular cues and reorient the growth axis. Evidence for the existence of two endocytic modes has been obtained in pollen tubes [29,43] and all the required molecular components have been identified [e.g. [9,34,44]]. The localization here reported for the pollen-specific SYP124 and its modulation by different signaling mechanisms fits this hypothesis. The changes observed on SYP124 distribution upon growth modulation possibly reflect a repositioning of the vesicle's docking machinery and highlight the importance of syntaxins in secretion and tip growth.

## Methods

### Plasmids and vectors

Lat52 pollen promoter (pLat52, [25]) was amplified from pGreen pLat52-GFP vector [45] with forward (5'-CCCCCC**AAGCTT**GTCGACATACTCGACTCAGAA-3') and reverse (5'-CCCCCC**CCATGG**TTGATTATAATGAAATAGCCTTTTTA-3') primers carrying *Hind*III and *Noc*I restriction sites (underlined) respectively. The resulting DNA fragment was used to replace 35S promoter (PCaMV35S) in plasmid GFP-122F [23], resulting in GFP-AtSYP122F. GFP-AtSYP121F was similarly constructed; AtSYP121 (AT3G11820, The Arabidopsis Information Resource) was amplified with forward (5'-GA**GTCGACC**ATGAACGATTTGTTTTCCAGCTCATTCTCTCGC-3') and reverse (5'-CGAAACTCAACTTCAA**CTGCAG**CTTCAACGCAA-3') primers carrying *Sal*I and *Pst*I restriction sites (underlined).

AtSYP124 cDNA (AT1G61290, The Arabidopsis Information Resource) was amplified with forward (5'**GTCGAC**CATGAATGATTTATTCTCTAGTTCGTTC-3') and reverse (5'-**CTGCAG**TCACTTCAACATGAGCATGATATGAGG-3') primers carrying *Sal*I and *Pst*I restriction sites (underlined) respectively. The resulting DNA fragment was used to replace the *Sal*I-*Pst*I fragment in plasmid pSGFP5K [46]. After digestion with *Nde*I and *Eco*RI the resulting DNA fragment was used to replace the corresponding fragment in plasmid GFP-AtSYP122F, resulting in GFP-AtSYP124F fusion coding sequence controlled by the pLat52.

GFP-AtSYP122F was amplified with forward (5'-GC**CCATGG**CCCCCCATGAACGATCTTCTCTCCGGC-3') and reverse (5'-AGC**CTGCAG**CAAAATGGCAAATCAAGTCCA-3') primers, resulting in 122T (AtSYP122ΔC287-R341) coding sequence carrying *Nco*I and *Pst*I restriction sites (underlined) respectively. The resulting DNA fragment was used to replace the *Nco*I-*Pst*I fragment in plasmid GFP-AtSYP122F resulting in 122T coding sequence controlled by the pLat52. Construct 124T was similarly obtained but amplifying GFP-AtSYP124F with forward (5'-GC**CCATGG**CCCCCCATGAATGATTTATTCTCTAGTTC-3') and reverse (5'-A**CTGCAG**ATCAAGTCCATTTCCTCGAGCTCTT-3') primers carrying *Nco*I and *Pst*I restriction sites (underlined) respectively, resulting in AtSYP124ΔC257-K283 coding sequence controlled by the pLat52.

pLat52:Rab11DN was constructed using the forward (5'-CCCCCCAAGCTT**GTCGAC**ATACTCGACTCAGAA-3') and reverse (5'-CGC**GGATCC**ATGGTTGATTATAATGAAATAGCCTTTTTATAG-3') primers carrying *Sal*I and *Bam*HI restriction sites (underlined) respectively. The resulting DNA fragment was used to replace the *Xho*I-*Bam*HI fragment - PCaMV35S - in plasmid Rab11S22/27N [23] resulting in Rab11S22/27N coding sequence controlled by the pLat52.

Construct PIP5K4 (pLat52-PIP5K4) was generated as described previously [27].

### Plant material

*Nicotiana tabacum *(cv Petit Havana SR1) plants were grown in a greenhouse under standard conditions. Mature pollen was collected and germinated in culture as previously [27].

### Transient transformation and GFP imaging in tobacco pollen tubes

A helium-driven PDS-1,000/He particle delivery system (BioRad) was used for the biolistic transformation of tobacco pollen as described by [27]. For single plasmid expression 2 μg of plasmid DNA was used per sample; for co-expression 2 μg of each plasmid DNA was used (unless stated otherwise). Pollen grains were then allowed to germinate and transferred onto cover slips for microscopic analysis. Cells expressing the construct(s) were followed by confocal laser scanning microscopy (CLSM) 5 to 9 hours after transformation.

Thin time-course optical sections (~2 μm thick) were acquired with a Leica SP-E CLSM using <20% laser intensity and operating in the mode 512 × 512, 400 Hz (~1/4 sec per frame). A ×20 Plan Apo dry objective (NA = 0.75) or a ×40 Plan dry (NA = 0.85) (Leica) were used. For quantification purposes, gain and offset settings were kept constant so that the average background pixel intensity was between 0 and 10 and the fluorescent signal coming from the cells was between 60 and 220 (0-255 scale for 8 bit images). Co-labelling with FM4-64 was performed as described before [13,27,30] with 2 μM of the FM solution (Molecular Probes) prepared in pollen tube growth medium and observed 5-10 min after labelling.

### Drug treatments

20 μM Gadolinium chloride (Sigma) and 3,4,5-trimethoxybenzoic acid 8-(diethylamino)octyl ester (TMB-8, Sigma) were prepared in liquid pollen growth medium (stock solution - 100 μM) and let to diffuse through the ~1-2 mm thick agarified (0.8%) growth medium. Upon inhibition of growth (~1 min), fresh growth medium was used to wash-out the inhibitors. This method minimizes any side perturbations to pollen tubes that could affect imaging settings and conditions (e.g. change of focus, mechanic shock, etc).

### Data analysis

Numerical data extraction was performed using Image-Pro Plus 6.0 software (Media Cybernetics). Fluorescence measurements of the pollen tube cytoplasm and/or plasma membrane correspond to medium fluorescence intensity in the areas depicted in the figure diagrams. Fluorescence was quantified in terms of average pixel intensity and statistically analysed with a t-student test (2-tailed distribution). Variations of >5% were considered to be meaningful.

Except where mentioned, numerical data in figures correspond to single cell analysis of typical experiments and not to summary statistics. This is because there is a significant degree of variability at a biological level but also at a technical one; even minor changes in the degree of expression, disturbance on drug addition, and responsiveness of the cell can play a role in the extent of cellular response [27,40].

## Abbreviations

[Ca^2+^]_c_: cytosolic free calcium; CLSM: confocal laser scanning microscopy; *PIP2: *phosphatidylinositol-4,5-bisphosphate; *PIP5K4: *phosphatidylinositol-4-monophosphate 5-kinase; *PM: *plasma membrane; *SYP: *syntaxin in plant (referring to all Q-SNAREs with a trans-membrane domain); TMB: 3,5,3',5'-tetramethylbenzidine-HCl

## Authors' contributions

RM conceived the study and prepared the manuscript. PAS carried out the imaging experiments and performed quantification. PAS, RUR, CR and GDS made the molecular constructs. All authors discussed the results, read and approved the final manuscript.

## Supplementary Material

Additional file 1 **AtSYP121, SYP122 and SYP124 sequences multiple alignment performed by Clustal X (1.83)**. An orange box overlays the C-terminal trans-membrane domain where homology is low and which corresponds to the deletion proposed in the soluble SYPs DN mutants 121T, 122T and 124T. A blue box overlays the N-terminal sequence to evidence also the low homology in this domain. Click here for file

Additional file 2 **Confocal imaging of a *N. tabacum *pollen tube expressing GFP-AtSYP124 and co-labelled with 2 μM FM4-64**. Scale bar = 10 μm. **A**: Optical section showing GFP-SYP124 distribution ~6 h after transformation. **B**: FM4-64 distribution after ~5 minutes staining. **C**: Merge of GFP and FM4-64 fluorescence highlights the co-localization at the plasma membrane flanks, 10-25 μm behind the apex. Click here for file
