# Supplementary figures and images for: Asymmetric localization of Arabidopsis SYP124 syntaxin at the pollen tube apical and sub-apical zones is involved in tip growth
Source: BMC Plant Biol. 2010 Aug 18;10:179. doi: 10.1186/1471-2229-10-179 (PMC3095309; doi:10.1186/1471-2229-10-179)

## Slide 1
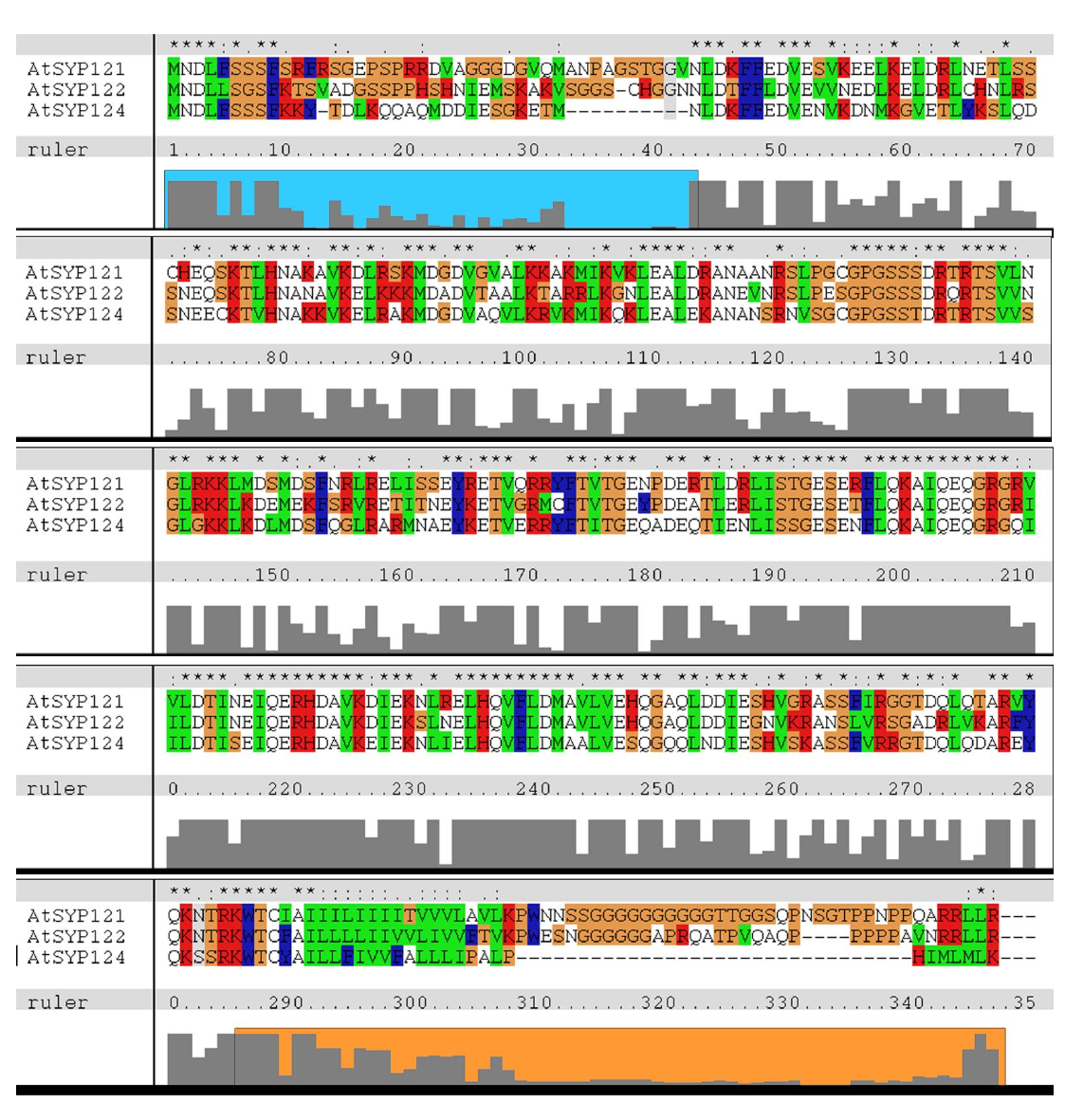

Supplement: Additional file 1 — AtSYP121, SYP122 and SYP124 sequences multiple alignment performed by Clustal X (1.83). An orange box overlays the C-terminal trans-membrane domain where homology is low and which corresponds to the deletion proposed in the soluble SYPs DN mutants 121T, 122T and 124T. A blue box overlays the N-terminal sequence to evidence also the low homology in this domain. [file 1471-2229-10-179-S1.PPT]

## Slide 1
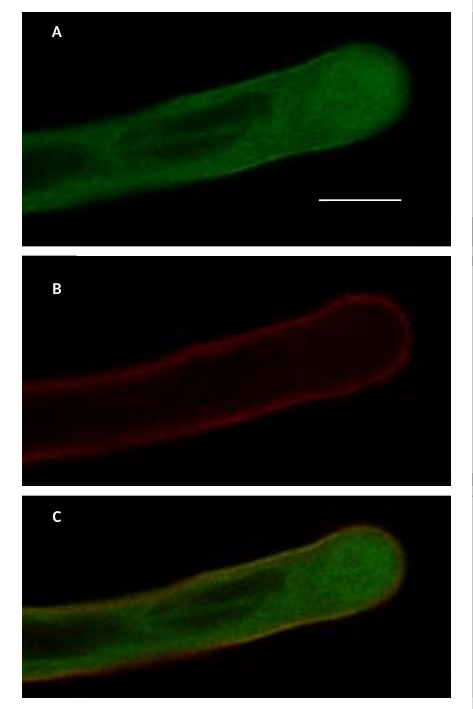

A
B
C

Supplement: Additional file 2 — Confocal imaging of a N. tabacum pollen tube expressing GFP-AtSYP124 and co-labelled with 2 μM FM4-64. Scale bar = 10 μm. A: Optical section showing GFP-SYP124 distribution ~6 h after transformation. B: FM4-64 distribution after ~5 minutes staining. C: Merge of GFP and FM4-64 fluorescence highlights the co-localization at the plasma membrane flanks, 10-25 μm behind the apex. [file 1471-2229-10-179-S2.PPT]
